# The neurotoxic effect of isoflurane on age‐defined neurons generated from tertiary dentate matrix in mice

**DOI:** 10.1002/brb3.1949

**Published:** 2020-11-17

**Authors:** Xin‐Li Xiao, Jing‐Tao Wu, Han‐Ze Zhang, Yi‐Di Wang, Jing‐Qiao Zhang, Le‐Fan Liu, Yu‐Chen, Min‐Li, Peng‐Bo Yang, Xiao‐Lin Wu, Jian‐Xin Liu

**Affiliations:** ^1^ Department of Human Anatomy, Histology and Embryology School of Basic Medical Sciences Xi'an Jiaotong University Health Science Center Xi'an China; ^2^ Institute of Neuroscience Translational Medicine Institute Xi'an Jiaotong University Health Science Center Xi'an China; ^3^ Zonglian College Xi'an Jiaotong University Health Science Center Xi'an China; ^4^ School of laboratory Medicine Hubei University of Chinese Medicine Wuhan China; ^5^ Institute of Neurobiology School of Basic Medical Sciences Xi'an Jiaotong University Health Science Center Xi'an China

**Keywords:** anesthetics, isoflurane, neurotoxicity, tertiary dentate matrix

## Abstract

**Introduction:**

Recent animal studies showed that isoflurane exposure may lead to the disturbance of hippocampal neurogenesis and later cognitive impairment. However, much less is known about the effect of isoflurane exposure on the neurons generated form tertiary dentate matrix, even though a great increase of granule cell population during the infantile period is principally derived from this area.

**Methods:**

To label the new cells originated from the tertiary dentate matrix, the mice were injected with BrdU on postnatal day 6 (P6). Then, the mice were exposed to isoflurane for 4 hr at 1, 8, 21, and 42 days after BrdU injection, and the brains were collected 24 hr later. The loss of newly generated cells/neurons with different developmental stage was assessed by BrdU, BrdU + DCX, BrdU + NeuN, or BrdU + Prox‐1 staining, respectively.

**Results:**

We found that the isoflurane exposure significantly decreased the numbers of nascent cells (1 day old) and mature neurons (42 days old), but had no effect on the immature (8 days old) and early mature neurons (8 and 21 days old, respectively).

**Conclusion:**

The results suggested isoflurane exposure exerts the neurotoxic effects on the tertiary dentate matrix‐originated cells with an age‐defined pattern in mice, which partly explain the cognitive impairment resulting from isoflurane exposure to the young brain.

## Graphical abstract

Isoflurane exposure exerts neurotoxic effects on the tertiary dentate matrix‐originated cells with an age‐defined pattern in mice. The isoflurane significantly decreased the numbers of nascent cells and mature neurons driven from the tertiary dentate matrix.

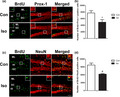

## 1 INTRODUCTION

The safety of anesthetics has been questioned since accumulating evidences from rodents and nonhuman primates have indicated that anesthetics exposure, especially in the young brain, might result in subsequent neurobehavioral abnormalities including cognitive impairment (Andropoulos, 2018; Drobish et al., 2016; Ju et al., 2019; Nagy et al., 2015; Sun et al., 2014; Wang et al., 2016). Anesthetics induced alteration of hippocampal neurogenesis was strongly suggested to potentially contribute to the subsequent impairment in developing brain, as rodent models showed numerous deficits in behavioral tasks of learning that were hippocampal‐dependent (Huang et al., 2016; Jiang et al., 2016; Tung et al., 2008; Wang, Lu, et al., 2018).

In the rodent hippocampus of early postnatal period, the granular cells in the dentate gyrus (DG) are made up of a heterogeneous population of neurons regarding their original sites, that is, primary dentate neuroepithelium, secondary, and tertiary dentate matrix (Altman & Bayer, 1990). A great increase in granule cell population during the infantile and juvenile periods is principally derived from the tertiary dentate matrix (Schlessinger et al., 1975). Furthermore, the neurons generated from different sites were found to exhibit respective survival phenotypes (Ciric et al., 2019), suggesting their response to the anesthetics exposure may be not identical. Actually, even for the cells with the same origin site, the extent of cell death following the anesthetics exposure still depends on the developmental stage of these cells (Erasso et al., 2013). Consequently, detailed analysis of cell loss in original site‐ and age‐defined neurons undergoing anesthetics exposure is critical to understand the mechanism of developmental anesthetic neurotoxicity in the brain.

Severe neurotoxicity has been reported against isoflurane, one of commonly used anesthetics (Andropoulos, 2018; Drobish et al., 2016; Wang et al., 2016). In this study, we used the thymidine analog BrdU to label the newly generated cells in the DG neurons at peak of the DG development stage, that is, postnatal day 6, during which the new cells are generated from the tertiary dentate matrix. Using specific markers of neuronal differentiation and maturation, we assessed the effects of isoflurane exposure on the cells originated from the tertiary dentate matrix with different developmental stages.

## 2 MATERIALS AND METHODS

### 2.1 Animals

The animal protocol was approved by the Experimental Animal Center of Xi'an Jiaotong University Health Science Center (certificate no. 22‐9601018), and all mice were treated in strict accordance with APS/NIH guidelines. Experimental agreement was approved by the Animal Care and Use Regulation of Xi'an Jiaotong University Health Science Center. The Swiss mice were housed with free access to food and water on a 12‐hr light/dark cycle. The birth date of the pups was considered to be P1.

### 2.2 BrdU injection

On P6, the pups were received i.p. injections of 5‐bromo‐2′‐deoxyuridine (BrdU, Sigma, B5002, St. Louis, Missouri) at a dose of 50 mg/kg for three times with a intervals of 4 hr. The average weight of mice on P5 was 4.12 g. BrdU solution was dissolved in 0.9% saline and 0.007 N NaOH at 5 mg/ml and stored at −20°C. After injection, neonates were returned immediately to the cage with the mother.

### 2.3 Isoflurane exposure

At 1, 8, 21 and 42 days after BrdU injection, that is, at P7, P14, P27, and P48, mice were randomly divided into isoflurane‐exposed and control groups. The isoflurane‐exposed group received 1.5% isoflurane (Lunan Bert Pharmaceutical Group Corporation, Shanghai, China) in 1 L per minute of gas mixture (50% O_2_ and 50% air) in a plastic chamber for 4 hr. Regimen of isoflurane treatment was selected based on our preliminary experiment and previous studies (Erasso et al., 2013; Wang, Wu, et al., 2018). The size of the anesthesia chamber in the study was 20 × 20 × 10 cm. The chamber was kept in a homoeothermic incubator to maintain the experimental temperature at 37°C. We observed responses of mice to tail clamping every 15 min under anesthesia. Respiratory rate, skin color, and body movement were observed carefully. Meanwhile, control mice were exposed to the gas mixture without isoflurane at the same flow rates for 4 hr. The concentrations of oxygen, carbon dioxide, and isoflurane in the chamber were continuously monitored using a DrägerVamos Plus infrared gas analyzer (Drägerwerk AG, Germany). The mice exposed to isoflurane at P7 and P14 were returned to their mother mice, and other groups were put back the cages after exposures.

### 2.4 Tissue preparation and immunohistochemistry

Twenty‐four hours after the exposure to isoflurane or control gas, that is, at P8, P15, P28, and P49, five mice of each group (anaesthetized by 0.6% pentobarbital) were perfused transcardially with 0.9% saline 8 min, followed by 100 ml 4% paraformaldehyde in 0.1 M phosphate buffer (PB) (pH 7.4) for 10 min. The brains were removed and postfixed in 4% paraformaldehyde at 4°C for 24 hr. The brains were then put in 30% sucrose dissolved by 0.1 M PB for overnight. Serial coronal sections at 40 µm thickness were cut in a cryostat (Leica, Germany). Serial sections were transferred to different wells of a 24‐well tissue culture dish for immunocytochemical study. All sections of the entire DG without interruption were obtained in four (P8) or six wells (P15, P28, and P49) for immunocytochemical analysis, and each well consisted of 6–8 sections of the DG.

For immunofluorescence double‐labeling, the sections were incubated in 2 N HCl for 30 min at 37°C for denaturation of the DNA, and then, the sections were rinsed in 0.1 M boric acid (pH 8.5) for 10 min at room temperature, rinsed 3 times with 0.1 M PBS for 10 min. Then, sections were incubated in blocking solution (10% goat serum in 0.3% Triton X‐100 PB‐buffered saline) for 1 hr at room temperature. Sections were incubated overnight at 4°C with the primary antibodies: rat monoclonal anti‐BrdU antibody (1:1,000, Abcam, UK), rabbit monoclonal anti‐DCX antibody (1:500, Abcam, UK), rabbit monoclonal anti‐NeuN antibody (1:3,000, Abcam, UK), and rabbit monoclonal anti‐Prox‐1 antibody (1:500, Abcam, UK). On the next day, sections were placed in secondary antibody conjugated to FITC (rabbit anti‐rat, GeneTex, USA) and Cy3 (goat anti‐rabbit, GeneTex, USA) for 4 hr. Sections were incubated with PBS instead of the primary antibodies as negative controls. BrdU‐, BrdU + DCX‐, BrdU + NeuN‐, and BrdU + Prox‐1‐positive cells were observed by fluorescence microscopy (BX51, Olympus, Tokyo, Japan).

### 2.5 Cell counting

Cell counting was done using the Image‐Pro Plus system (Media Cybernetics, Silver Spring, MD, USA) interfaced through an Olympus microscope. The BrdU‐positive cells were manually counted in the target areas by using every 4th (P8) or 6th (P15, P28, and P49) section of each animal (6–8 sections/animal). Then, the derived number was multiplied by 4 or 6 for each animal to estimate the total number of BrdU‐positive cells in the bilaterally target areas (Iqbal et al., 2019). For cell counting of BrdU‐positive cells in P8 DG, where the BrdU‐positive cells were fill with hilus, BrdU + NeuN double staining were done to show the GC and hilus clearly with NeuN signal. The double‐labeled cells were determined and counted with a fluorescence microscope at a magnification of 200×. The procedure for calculating the absolute number of double‐labeled cells per DG for each animal was the same as that for BrdU‐positive cells' counting.

### 2.6 Statistics

All data used in this study were presented as mean ± standard error of mean (*SEM*) and were analyzed via a two‐sample *t* test followed by Dunnett's *t* test. All analyses were performed with Prism 5 software (GraphPad, USA). Values of *p* < .05 were considered significant.

## 3 RESULTS

### 3.1 The effect of isoflurane on the nascent cells from the tertiary dentate matrix

**Figure 1:**
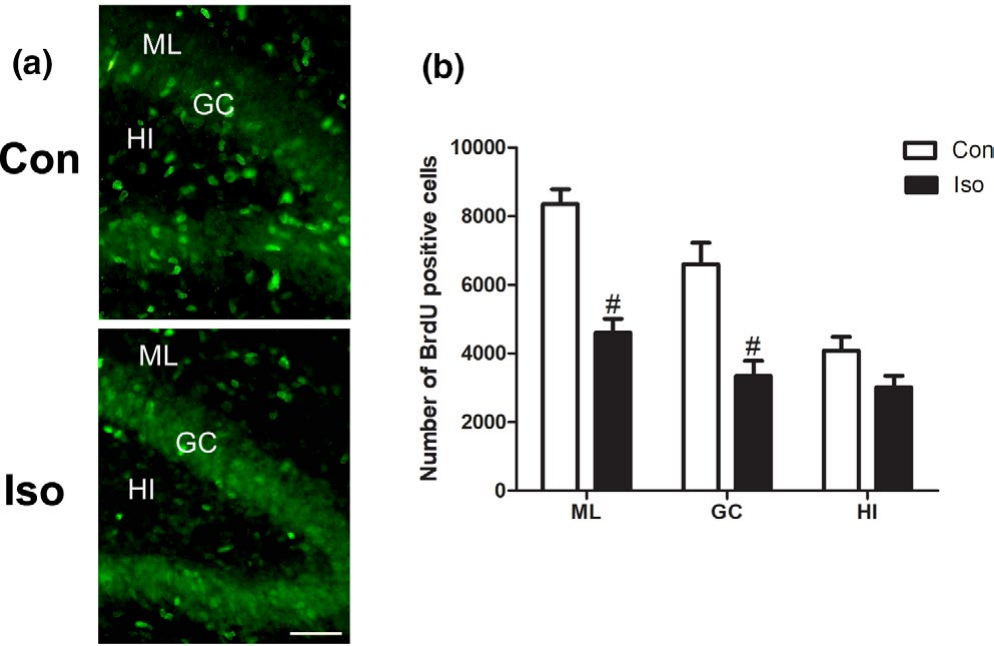
BrdU staining: the effect of isoflurane on the nascent cells. (a) There are significantly more BrdU cells in the ML, GC of DG in the control group than isoflurane‐treated group. (b) Quantitative analysis of the number of BrdU‐positive cells between the two groups. All data are presented as mean ± *SEM*.*n* = 5 for each group. #*p *˂ 0.01 versus control. Scale bar = 100 µm

To detect the effect of isoflurane on the nascent cells (1 day old), we examined the total number of BrdU‐positive cells in the molecular layer (ML), granular cell layer (GC), and hilus (HI) of DG in the isoflurane‐treated and control animals. BrdU‐positive cells were distributed in all layers of the DG (Figure 1a). There were significantly more BrdU cells in the ML, GC of DG in the control group compared to the isoflurane‐treated group (Figure 1b, *t* = 6.390, *p* = .0002 and *t* = 4.218, *p* = .0029, respectively). However, no significant difference was found for the number of BrdU cells in the HI (Figure 1b) (*t* = 2.031, *p* = .0767).

### 3.2 The effect of isoflurane on the immature neurons generated from the tertiary dentate matrix

**Figure 2:**
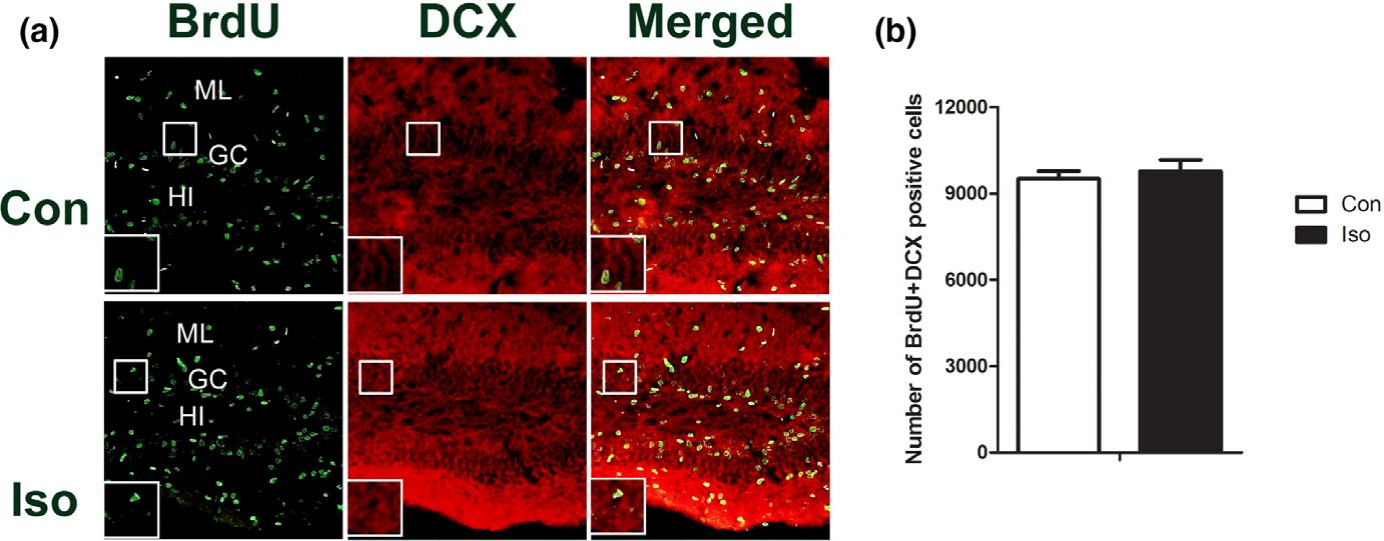
Double‐labeling staining of BrdU + DCX: the effect of isoflurane on the immature neurons. (a) Representative double immunofluorescence images of BrdU + DCX‐positive cells in the DG. (b) Quantitative analyses of BrdU + DCX‐positive cells in the DG show that there is no significant difference on the number of double‐labeling cells between the two groups. All data are presented as mean ± *SEM*.*n* = 5 for each group.*p ˂ 0.05 versus control. Scale bar = 100 µm

To determine the effects of isoflurane on the immature neurons (8 days old) in neonatal mice, BrdU + DCX fluorescent staining were performed and the number of double staining in the GC was counted. BrdU‐positive cells were distributed in all layers, while DCX‐positive cell bodies were in the GC and its dendritic processes were in the ML (Figure 2a). There was no significant difference of double staining cells between the control and isoflurane‐treated groups (Figure 2b) (*t* = 0.5504, *p* = .5970).

### 3.3 The effect of isoflurane on the early mature neurons generated from tertiary dentate matrix

**Figure 3:**
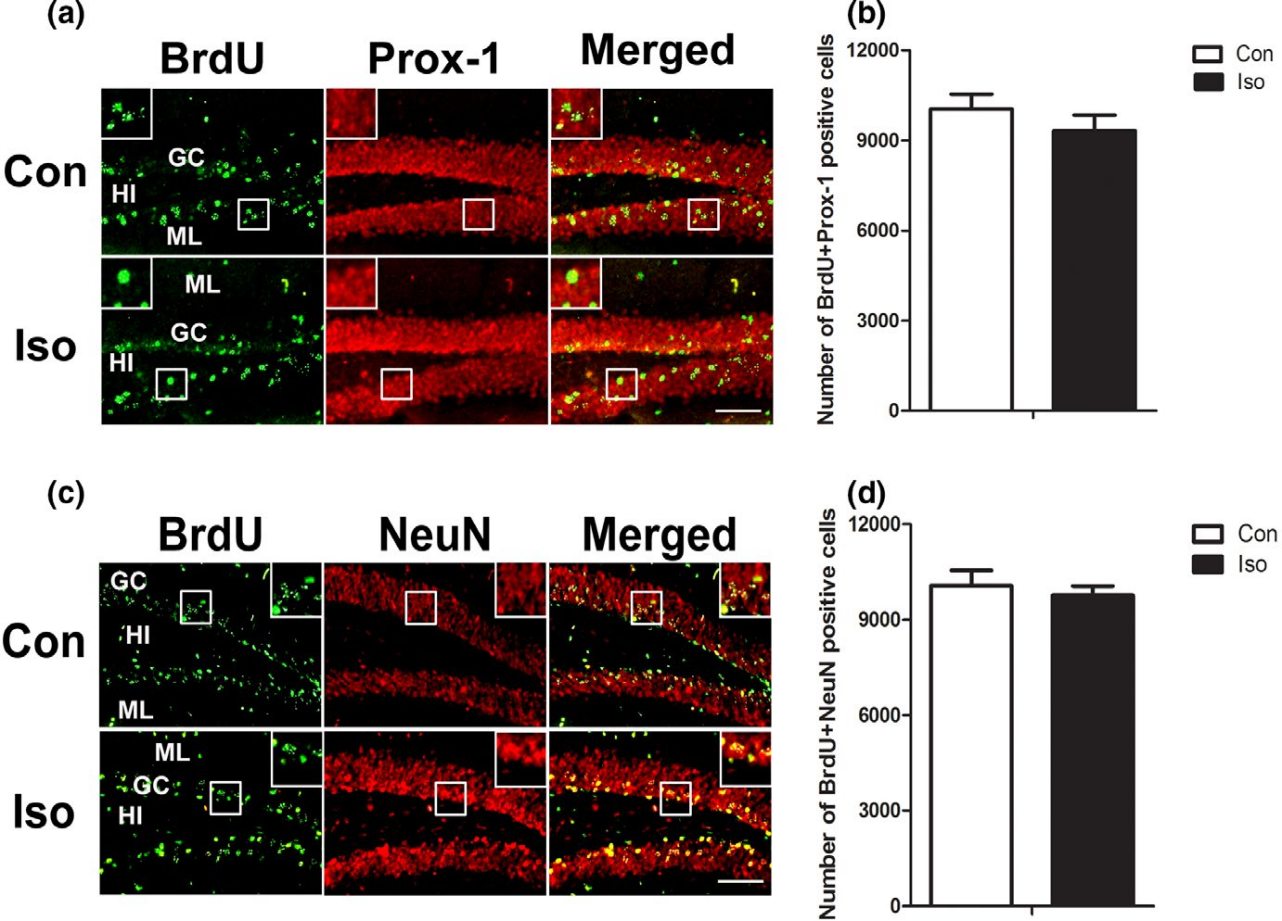
Double‐labeling staining of BrdU + Prox‐1 and BrdU + NeuN: the effect of isoflurane on the early mature neurons. a and c: Representative double immunofluorescence staining images of BrdU + Prox‐1‐ and BrdU + NeuN‐positive cells in DG. b and d: Quantitative analyses of double‐labeling positive cells do not find any difference on the number of double‐labeling cells between the two groups. All data are presented as mean ± *SEM*.*n* = 5 for each group. *p ˂ 0.05 versus control. Scale bar = 100 µm

To detect the effects of isoflurane on new cells undergoing early mature stage (21 days old), BrdU + Prox‐1 and BrdU + NeuN fluorescent staining were performed and the number of double staining in the GC was counted. BrdU‐positive cells were distributed in all layers of the DG, particularly in the GC (Figure 3a,c). BrdU + Prox‐1 immunostaining is shown in Figure 3a, and BrdU + NeuN immunostaining is shown in Figure 3c. Quantitative study indicated no significant difference of BrdU + NeuN or BrdU + Prox‐1 double staining cells between the control and isoflurane‐treated groups (Figure 3b,d) (*t* = 1.007, *p* = .3436 and *t* = 0.3305, *p* = .7472, respectively).

### 3.4 The effect of isoflurane on the mature neurons generated from the tertiary dentate matrix

**Figure 4:**
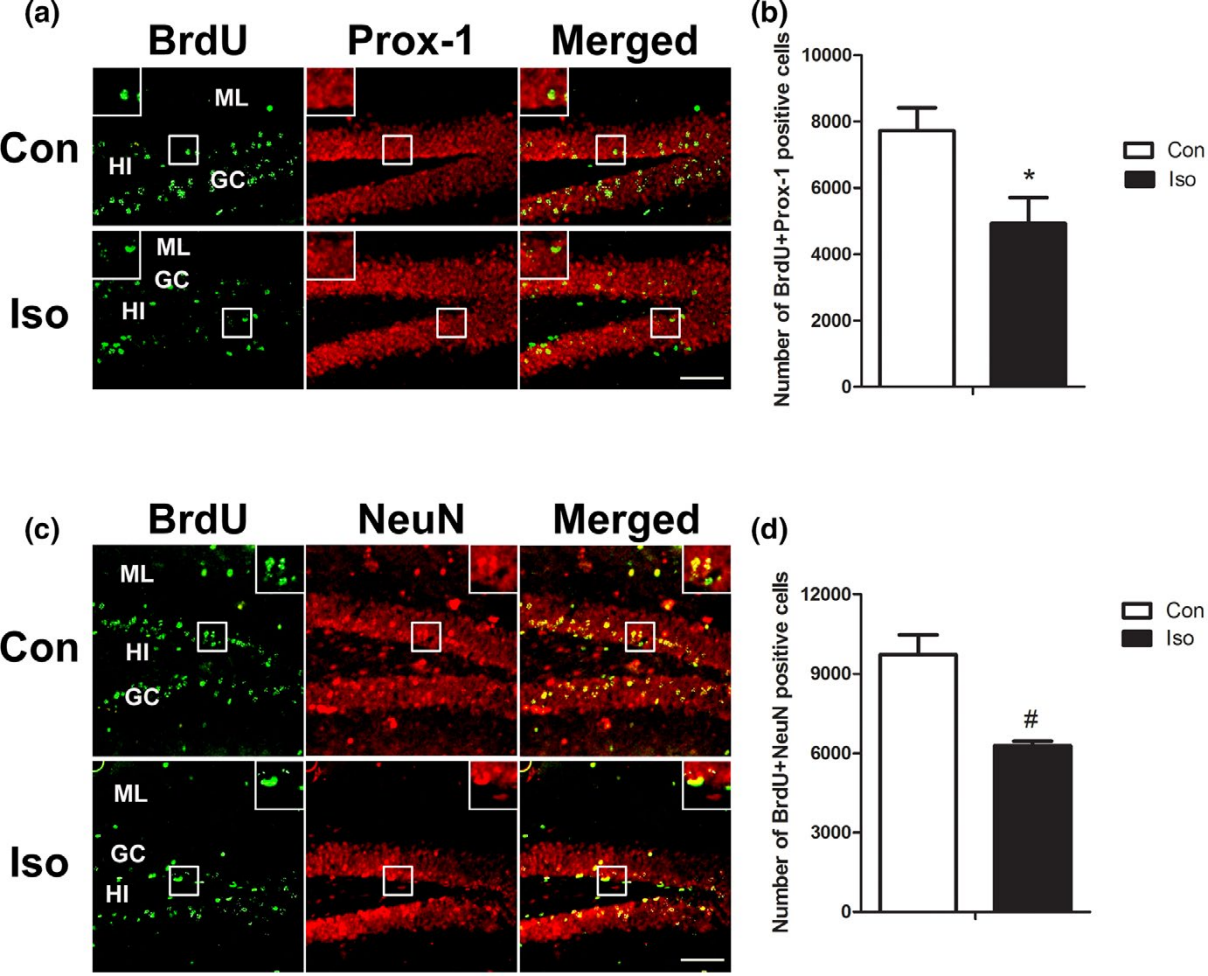
Double‐labeling staining of BrdU + Prox‐1 and BrdU + NeuN: the effect of isoflurane on the mature neurons a and c: Representative double immunofluorescence staining images of BrdU + Prox‐1‐ and BrdU + NeuN‐positive cells in the DG. b and d: Quantitative analyses of double‐labeling positive cells show that the number of double‐labeling cells reduced significantly in the isoflurane‐treated group compared with the control group. All data are presented as mean ± *SEM*.*n* = 5 for each group.*p ˂ 0.05 and #*p* < .01 versus control. Scale bar = 100 µm

Next, we tested the effects of isoflurane on the mature neurons generated from the tertiary dentate matrix by performing BrdU + Prox‐1 and BrdU + NeuN fluorescent staining. BrdU‐positive cells were still distributed in all layers, and most cells were in the GC (Figure 4). The number of BrdU + Prox‐1 double staining cells decreased significantly in the isoflurane‐treated group compared to the control group (Figure 4b) (*t* = 2.686, *p* = .0228). In order to confirm the result, the number of BrdU + NeuN positive cells was also counted (Figure 4d), fewer double staining cells were observed in the isoflurane‐treated group when compared with the control group (Figure 4b,d) (*t* = 4.463, *p* = .0021).

## 4 DISCUSSIONS

While there is abundant evidence that anesthetics act to suppress subgranular zone (SGZ)‐derived neurogenesis in young and adult rodents, the anesthetic effects on the new neurons generated from the dentate matrix in the early postnatal period remain under‐examined (Erasso et al., 2013; Lin et al., 2013; Stratmann et al., 2010; Tong et al., 2019; Wang, Wu, et al., 2018). Drobish et al. (2016) labeled the proliferative cells by BrdU on P1, during which the new cells are generated from the second dentate matrix, and found that the number of BrdU‐positive cells significantly decreased in the DG at P7 following isoflurane exposure at P2, suggesting isoflurane exposure exerted neurotoxic effect on nascent cells or decreased their survival abilities. Wang, Lu, et al. (2018) had exposed rats to isoflurane on P7, and BrdU was administered both 4 hr before and after isoflurane exposure. Hence, their results mixed the effects of isoflurane on nascent cells and cell proliferation in the tertiary dentate matrix. In addition, labeled BrdU during and after isoflurane exposure with P7 rats, Stratmann and co‐workers found a decreased progenitor proliferation until at least 5 days after isoflurane exposure (Stratmann et al., 2009). Mixing the effects on the proliferation and survival abilities, these studies mentioned above have not draw a clear profile of the neurotoxic effect of anesthetics on the age‐defined cells driven from the dentate matrix.

To clarify the effects of isoflurane exposure on age‐defined neurons generated from the tertiary dentate matrix, isoflurane exposure was performed during different developmental stages of neurons generated from the tertiary dentate matrix, that is, nascent cells (1 day old and labeled with BrdU), immature stage (8 days old, labeled with BrdU + DCX), early mature stage (21 days old, labeled with BrdU + Prox‐1 and BrdU + NeuN) and mature stage (42 days old, labeled with BrdU + Prox‐1 and BrdU + NeuN), respectively (Brandt et al., 2003). The neurotoxic effect of isoflurane on age‐defined cells was assessed 24 hr after isoflurane exposure. In the present study, the mice were received 1.5% isoflurane exposure for 4 hr because the studies employing this regime to look at the behavioral effect of isoflurane have reported deficits in learning and memory in mice and rats without leading to significant metabolic abnormalities such as arterial blood gases and glucose levels (Loepke et al., 2009; Wang et al., 2016).

We found that the number of nascent cells (1 day old) significantly reduced in the ML and GC of DG at 24 hr after isoflurane exposure, indicating the recently postmitotic cells are particularly vulnerable to the anesthetic insult. Our present study focused on the cells derived from the tertiary germinal zone, rather than on the cells from the SGZ, possibly accounting for some “contradictory” results from other groups who found isoflurane had no effect on 1‐day‐old newly born cells in adult mice (Jiang et al., 2016). Because BrdU‐positive cells with 1 day old were fate‐uncommitted neural progenitors, further study should be designed to examine detail effects of isoflurane on different fate nascent cells generated from the tertiary dentate matrix. In addition, caspase‐3 can be activated in the newly generated cells several hours after isoflurane exposure (Drobish et al., 2016; Wang, Lu, et al., 2018). We hence rationally attributed the decrease BrdU‐positive cells to isoflurane‐induced apoptosis, although a direct apoptosis assessment is not included in our present study.

Because BrdU + DCX‐, BrdU + NeuN‐, and BrdU + Prox‐1‐positive cells were only located in the GC of DG, the number of the double‐labeled cells was just counted in this area. Our results indicated isoflurane exposure did not affect the immature (8 days old) and early mature (21 days old) stage of neurons. Similarly, Erasso et al. (2013) also observed that isoflurane had no effect on SGZ‐derived newly born neurons undergoing differentiation (8 days old) or maturation (21 days old) in the DG of 3‐month‐old rats. Together, these results may suggest that the newly born neurons undergoing differentiation and maturation may be more resistant to the isoflurane exposure regardless of their originations. Interestingly, the decreased number of new neurons with mature stage, that is, 42 days old, was demonstrated following isoflurane exposure. Ciric et al. (2019) suggested 17% of P6‐born DG neurons showed delayed cell death after reaching maturity in young adult, suggesting the tertiary dentate matrix‐derived mature neurons might be more sensitive to the insults such as isoflurane exposure.

The tertiary dentate matrix appears on day E22 and is replaced by SGZ between days P20 and P30 (Altman & Bayer, 1990). While many studies found isoflurane exposure to rats during infantile period caused delayed‐onset hippocampal‐dependent cognitive deficits (Chinn et al., 2019; Stratmann et al., 2009; Wang, Lu, et al., 2018; Wang et al., 2016; Zhu et al., 2010), the impairment of cognitive functions was not demonstrated following isoflurane exposure to adult animals (Altman & Bayer, 1990; Stratmann, Sall, Bell, et al., 2010; Walters et al., 2016; Zhu et al., 2010). Our finding that isoflurane exposure has neurotoxic effect on nascent and mature neurons derived from tertiary dentate matrix may, in part explain, the cognitive impairment resulting from isoflurane exposure to the young brain.

Our experiments showed effects of isoflurane exposure on age‐defined neurons generated from the tertiary dentate matrix. These results suggest a possible mechanism for the impairment of cognitive function reported after exposure to isoflurane. Clearly, questions regarding the effect of other anesthetics and underlying mechanisms remain to be elucidated.

## 5 CONCLUSION

When a great increase of granule cell population during the infantile period is principally derived from the tertiary dentate matrix, much less is known about the effect of isoflurane exposure on this population of neurons. Using specific markers of neuronal differentiation and maturation, we demonstrated that isoflurane exposure could exert a neurotoxic effect on the nascent cells and mature neurons driven from the tertiary dentate matrix, which may partly explain the cognitive impairment resulting from isoflurane exposure to the young brain.

## CONFLICT OF INTEREST

The authors have no conflict of interest to declare.

## AUTHOR CONTRIBUTIONS

Liu JX and Xiao XL designed the project. Xiao XL and Liu JX wrote the manuscript. Xiao XL, Wu JT, Zhang HZ, Wang YD, Zhang JQ, Liu LF, Chen Y, Li M, Yang PB, and Wu XL conducted and analyzed the experiments. All authors analyzed and discussed the results and commented on the manuscript.

### Peer Review

The peer review history for this article is available at https://publons.com/publon/10.1002/brb3.1949.

## Data Availability

The data that support the findings of this study are available from the corresponding author upon reasonable request.
